# Worldwide barriers of optimal surgical care provision in advanced ovarian cancer

**DOI:** 10.1002/ijgo.70033

**Published:** 2025-04-25

**Authors:** Shriya Varghese, Myong Cheol Lim, Robert Armbrust, Rene Pareja, Christina Fotopoulou

**Affiliations:** ^1^ Department of Surgery and Cancer Imperial College London London UK; ^2^ Center for Gynecologic Cancer Research Institute and Hospital, National Cancer Center Goyang Republic of Korea; ^3^ Department of Gynecology with Center for Oncological Surgery—Charité Berlin European Competence Centre for Ovarian Cancer Berlin Germany; ^4^ Department of Gynecologic Oncology Instituto Nacional de Cancerologia Bogotá Colombia; ^5^ Gynaecologic Oncology Imperial College Healthcare NHS Trust London UK

**Keywords:** barriers, challenges, disparities, global, ovarian cancer, surgery

## Abstract

Ovarian cancer (OC) remains one of the most challenging gynecological malignancies to cure, despite recent advances in treatment. Disparities in the diagnosis, management, and survival of OC exist worldwide and addressing them remains an ongoing challenge. The highest burden of OC is projected to be in women living in low‐ and middle‐income countries, where mortality rates are also disproportionately higher. Maximal effort cytoreduction paired with maximal effort systemic therapy followed by maintenance therapies remain the cornerstones of treatment for OC. Disparities are twofold: first, due to challenges with systemic therapy; and second, due to variations in surgical care, especially for advanced disease. While the goals of surgery remain unchanged, the radicality of cytoreductive resections and variation in practices worldwide have increased. The provision of surgical care for OC patients faces numerous challenges broadly categorized into three main areas: health system barriers; patient‐related barriers; and physician‐related barriers. Health system challenges include the lack of centralized cancer care, scarcity of resources, and inadequate funding. Patient‐related obstacles include disparities in patient education, comorbidities, socioeconomic factors, and underrepresentation of certain ethnicities in clinical trials. Physician‐related barriers encompass suboptimal surgical training, limited access to educational resources, inconsistent adherence to guidelines, limited use of a multidisciplinary team and overall differences in philosophy, ethos, and surgical tradition. Addressing and overcoming these barriers is essential to ensure equitable access to high‐quality surgical care for OC patients worldwide. The aim of the present review was to further explore these global challenges while also highlighting potential strategies to reduce disparities in women's health care.

## 1 INTRODUCTION

Ovarian cancer (OC) remains one of the most challenging gynecological malignancies to cure, despite continuous advances in its overall management. It is currently the eighth most common cancer among women worldwide, with an estimated annual incidence of 324 000 cases.^1^ By 2050, the incidence of OC is projected to increase by 55%, with mortality rates expected to follow a similar trend.^2^ As with any cancer diagnosis, it places a significant economic and health burden not only on patients and their families but also on healthcare systems and governments globally.^3^ Disparities in the diagnosis, management, and survival of OC continue to exist worldwide, with the “postcode lottery” having a large effect on patient outcomes.^4^ Addressing this remains an ongoing challenge. The highest burden of OC is projected to be observed in women living in low‐ and middle‐income countries (LMICs), where mortality rates are also disproportionately higher.^3^, ^5^ Disparities in OC outcomes apply on two levels: systemic and surgical management. These disparities can be attributed to limited or restricted access to novel agents, infrastructural support, modern imaging, molecular or genetic diagnostics, and availability of intensive care unit (ICU) beds, to name a few key issues.^6^

Maximal effort cytoreductive surgery, complemented with combined cytotoxic chemotherapy in either the neoadjuvant or adjuvant setting and followed by maintenance therapy, comprises the current mainstay treatment algorithm for OC.^7^ Total macroscopic tumor clearance remains the ultimate goal of any cytoreductive approach in an effort to increase survival; however, the limits of operability have significantly expanded with the radicality of resections continuously increasing.^7^, ^8^ Multivisceral resection techniques, such as splenectomy, paracardiac lymph node resections, pleural resections, gastrointestinal resections, and hepatobiliary procedures belong to the routine armamentarium of any accredited gynecological oncologist.^8^ Equally, the overall profile of the surgical candidates has shifted to older and more frail patients with more comorbidities who can be safely brought to surgery despite their high tumor burden.^9^ This is also supported by improvements in pre‐ and postoperative care now being offered to patients, including advances in interventional radiology techniques, such as insertion of percutaneous drains into the peritoneal or pleural cavity as well as nephrostomies. This significant evolution of holistic surgical care for OC has opened avenues for patients who would have been previously deemed as inoperable, contributing to improved collective survival rates. Nevertheless, the implementation of a maximal effort surgical approach and associated surgical practices vary broadly around the world. In the UK alone, the national OC Audit Feasibility Pilot demonstrated significant variations in surgical treatment received by patients based on geographic cancer alliances.^10^ They also found patients in alliances that are less likely to perform surgery, sustaining a lower than average 5‐year overall survival.^10^ Findings from The World Ovarian Cancer Coalition Every Woman Study (EWS) involving a large‐scale patient survey also highlighted this discrepancy.^11^ The results of this, consisting of 1531 responses from 44 countries, found that only 60% of patients were offered surgery as part of their OC management.^11^

The aim of the present review was to further explore these global challenges while also highlighting potential strategies to reduce disparities in women's health care.

## 2 HEALTH SYSTEM BARRIERS

### 2.1 Lack of centralized cancer care systems

Over the past decade, growing evidence has supported restructuring gynecological cancer care towards a centralization model to dedicated specialist centers. A specialist center manages high patient volumes, offering high expertise surgery led by gynecology oncologists and multidisciplinary team (MDT) support.^12^ A Cochrane review by Woo et al.^13^ has demonstrated that patients treated at specialist centers by gynecologic oncologists had a survival advantage over those treated at general hospitals. The UK and Scandinavian countries serve as models, having adopted that model of centralization as first in the world.^14^, ^15^ Even though increasing attempts are made towards that direction by international gynecological oncology societies such as the International Gynecologic Cancer Society (IGCS) and European Society of Gynecological Oncology (ESGO), most countries are yet to implement the centralized care model.^12^ Currently, the National Comprehensive Cancer Network guidelines recommend that all women with stage 1B to 4 OC receive care from a specialist gynecology oncologist.^16^ The Global Equality in Ovarian Cancer Care (GEOCC) survey launched by the IGCS revealed that nearly 20% of lower‐middle/low‐income (LMLI) clinicians reported that OC surgery is performed by general obstetricians and gynecologists not specialized in complex procedures.^6^ Findings from a patient survey found that only 50%–60% of OC patients received care in a specialist center in Germany.^11^ To explore further reasons behind why patients did not receive care from a specialist, Weeks et al.^16^ conducted a qualitative study interviewing patients who did not receive specialist care. From a patient's point of view, affordability, an apprehension to attend large tertiary centers, and greater trust in their local physician were reported as notable barriers for specialist care. However, the biggest obstacle for a patient obtaining specialist care were patients being unaware of the importance of being managed by a gynecology oncologist.^16^

The USA is frequently cited as lacking centralized care among high‐income countries with nearly 30% of OC patients in the US not receiving specialist care.^16^ A retrospective study by Bristow et al.,^17^ using the California Cancer Registry, evaluated survival outcomes for 11 865 patients with stage 3C or 4 OC. This study revealed that only a minority of patients received treatment at high‐volume centers by high‐volume physicians (4.3%), whereas 53.1% were treated at low‐volume centers by low‐volume physicians.^17^ Patients treated at high‐volume centers by high‐volume physicians demonstrated the best median survival outcomes, highlighting the importance of quality centers and specialized physicians.^17^

A major challenge to centralizing care is the lack of data on the organization of OC management in LMICs. To address this knowledge gap, the GEOCC project began with an expert opinion survey to capture international differences in OC care practices.^6^ A total of 1059 responses from clinicians in 115 different countries was obtained with 25% of responses from LMLI countries and 29% from upper‐middle income (UMI) countries.^6^ Overall, 40% of clinicians reported an absence of dedicated OC centers in their country. LMLI countries had significantly lower access to cancer centers compared to high‐income countries (odds ratio [OR] 0.6, 95% confidence interval [CI] 0.5–0.8; *P* ≤ 0.001) and both UMI (OR 0.6, 95% CI 0.4–0.8; *P* ≤ 0.001) and LMLI (OR 0.3, 95% CI 0.2–0.4 *P* ≤ 0.001) were less likely to have regional OC networks.^6^ In addition, LMLI countries reported a rate of absence of cancer registries (29.6%) that is double that of high‐income countries (14.2%).^6^ A universal move towards centralization of care could enable patients access to specialized surgical expertise, which can be critical for effective treatment and improving survival outcomes. Other potential challenges associated with centralizing care include cost of implementation, disruptions to existing patient care, the impact of adverse weather on travel, and the increased travel distances that some patients may face.^18^ Although centralization can lead to economies of scale once fully established, the initial costs of restructuring healthcare systems can be substantial.^19^ This approach may not be a viable option for many LMLI countries, where health expenditure as a percentage of gross domestic product per capita is often among the lowest.^20^

### 2.2 Scarcity of resources and infrastructural support

Physical resources and infrastructural support are key to the safe provision of high‐quality surgical care, as the entire medical community very soberly was reminded during the COVID‐19 pandemic.^21^ The COVID‐19 pandemic not only affected patient health outcomes but also imposed a significant financial burden on countries as they struggled to meet the demands of an overstressed health sector. The CovidSurg Gynaecologic Oncology Cancer study, a prospective international study, assessed the pandemic's effects on surgical practices for eligible gynecology oncology patients.^21^ Data from 3973 patients across 52 countries revealed that in approximately 21% of patients, the standard of care was adjusted and approximately 11% of patients experienced a significant delay in management, with those requiring more expert care, such as OC patients, being affected the most.^21^ Canceled surgeries were shown to be linked to significantly increased adverse oncologic outcomes, including disease progression and death.^21^

Similar to the above findings, the GEOCC survey identified inadequate resources as a major barrier to OC management across all income statuses.^6^ One of the major bottlenecks is access to ICU beds, which are crucial for postoperative recovery, particularly for patients with a high tumor burden undergoing extensive cytoreductive surgery, those with comorbidities, and naturally those facing complications. Even though the largest disparities are seen in low‐income countries, ICU bed shortages are a global issue.^22^ A systematic review addressing ICU availability in low‐income countries found that ICU beds are generally scarce and, if available, are typically located in larger cities, limiting broad accessibility.^22^ For example, Uganda, with a population of approximately 25 million women, has just one ICU bed per million people, while the Republic of Korea, with a similar population, has 106 ICU beds per million people.^22^, ^23^ Adding to the complexity, ICU availability does not necessarily guarantee access to the wider population, regardless of income. In Brazil, 86% of cities lack public ICU beds, restricting access to mainly those who can afford private care.^24^, ^25^ Another example of a restricted surgical resource for OC management is the availability of hyperthermic intraperitoneal chemotherapy (HIPEC). While the value of HIPEC is still being fully investigated with multiple ongoing trials, its use remains resource‐intensive and costly.^26^ Although a Korean study demonstrated HIPEC to be cost‐effective, its future widespread uptake will likely be restricted to countries with sufficient financial resources to support its implementation.^27^

Workforce shortages and limited access to operating time represent an additional challenge for surgical management.^6^, ^28^ In an expert survey of clinicians from seven high‐income countries, Norell et al.^28^ reported that inadequate staffing was identified as a barrier to OC management, particularly by clinicians from Ireland and Australia. Poor infrastructural support and accessibility to adequate resources have also been shown to be closely linked to a surgeon's willingness to undertake extensive surgery, a factor directly correlated with OC survival.^28^ Medicolegal aspects and penalization due to surgical complications, such as readmission and reoperation, lead even high‐volume surgeons to fatigue and practicing of a more defensive medicine approach to avoid detrimental sequalae to their careers as well as their sanity.^29^ A British Medical Association survey revealed alarming results in that regard.^30^ Nearly half of the surveyed doctors (45%) reported fear of making a medical error in their daily work, with 9 out of 10 (89%) indicating workplace pressure or lack of capacity being one of the main reasons.^30^ In addition, over half (55%) worry they will be unfairly blamed for errors that are due to systemic failings and pressures.^30^ Almost all doctors (93%) indicated that these systemic pressures negatively impact their ability to deliver safe patient care and, as a result, nearly half of doctors (49%) report practicing defensively.^30^

Adding to the complexity, resource limitation extends beyond the surgical management of OC to also include systemic therapy. Even when optimal cytoreduction is achieved, limited access to chemotherapy and maintenance treatments like poly(ADP‐ribose) polymerase inhibitors (PARPi) negatively impacts patient outcomes.^31^ This issue is particularly pronounced in LMLI countries where disparities are the greatest.^31^ A review by Nogueira‐Rodrigues et al.^31^ highlighted several barriers that contribute to the disparities in accessing systemic therapy in LMLI countries. These include long wait times for drug approvals by health authorities, high costs of agents like PARPi, limited access to drugs in the public sector, as well as limited opportunities for participation in clinical trials.^31^ While improving surgical resources should remain a key priority, an integrated approach that also improves access to systemic therapy is essential to truly improve OC care.

### 2.3 Funding limitations for OC and related research

While cancer accounts for a large proportion of healthcare expenditure globally, OC patients specifically are often less prioritized, limiting both financial and clinical support.^32^ A global analysis by Chen et al.^32^ assessing the economic costs of 29 cancers across 204 countries found breast cancer and colorectal cancer to be among the top three most impactful cancers while OC ranked 16th. Government healthcare expenditure is largely influenced by the economic burden of a disease within a population. This could explain why governments may deprioritize funding for OC.^32^ Political stability is another important factor disproportionately affecting healthcare expenditure in LMLI countries. In politically unstable countries, government spending is likely to be redirected to other areas, such as defense.^33^ For example, in 2019, Afghanistan allocated 24% of government spending towards defense and less than 6% was allocated to healthcare, with similar findings in Senegal and Mali.^33^ Such reallocations disproportionately affect health outcomes for patients in LMLI countries, further exacerbating disparities.

Funding limitations and reduced prioritization of OC are not restricted to direct clinical spending, but also affect the investment into research for OC. In the UK, public research funding for OC has declined by 29% over 8 years, dropping from £12.9 million in 2010–2011 to £9.2 million in 2019–2020.^34^ In the USA, OC is equally underfunded.^35^ Spencer et al.^35^ analyzed the National Cancer Institute funding distributions for cancers between 2007 and 2014 and calculated a mean funding to lethality score for different cancers. In their study, breast cancer received a funding to lethality score of 1.803 compared to just 0.097 for OC.^35^ Not only did OC have a lower than average score but the 3‐, 5‐, and 8‐year trends have also shown a decline in OC's funding to lethality score.^35^ Similarly, McIntosh et al.^36^ reported that global cancer research funding for OC is misaligned with its burden, with much lower levels based on disability‐adjusted life years, mortality, and years lived with disability compared to other cancers. In addition, less than 10% of overall cancer research funding is directed towards metastatic disease, which is a common challenge in OC that complicates outcomes.^36^ This chronic underfunding creates a significant barrier to advancing, refining, and individualizing proper care for OC patients.

## 3 PATIENT‐RELATED BARRIERS

### 3.1 Patient education

A patient's understanding of their disease is essential for empowering them to make informed decisions and staying engaged in treatment, both of which can contribute to improved outcomes.^37^ The results from the EWS survey found that 69.1% of patients had either not heard about OC or known anything about the disease before their diagnosis.^11^ In addition, once informed about the diagnosis, 53.1% of patients reported not receiving sufficient information about the condition.^11^ Of the patients, 14.3% reported that the doctor informing them of their diagnosis spent less than 5 minutes with them during that interaction.^11^ Elshami et al.^38^ conducted a national cross‐sectional study examining the time taken by Palestinian women with OC to seek medical attention after symptom onset, as well as the barriers encountered in this process. Their findings revealed that patients with a greater awareness of OC symptoms, specifically recognizing 10 out of their proposed 11, were associated with a higher likelihood of seeking medical advice within just 1 week.^38^ Furthermore, emotional barriers, such as fear of diagnosis, were found to be the most common obstacle for patients seeking medical attention.^38^ Health education empowers patients to seek timely medical attention and to make informed decisions about all aspects of their care, including whether to undergo surgery and when to seek a second opinion if surgery is not initially offered. It may also help patients overcome the fear that may be associated with a potential cancer diagnosis. Furthermore, health education is especially critical for OC, where timely diagnosis and appropriate surgical intervention can impact survival. Patient education not only improves individual outcomes but also fosters avenues for advocacy, enabling those with OC to raise awareness, support research, and drive change that benefits women around the world. Globally, women's cancers are often burdened by significant stigma, which is exacerbated in LMLI countries by cultural beliefs and gender inequality.^39^ Notably, 64% of physicians in UMI and 74.8% in LMLI countries reported the lack of patient advocacy groups for OC cancer.^6^ Ensuring strong advocacy for these patients should be a critical international priority for the gynecology oncology community.

### 3.2 Patient comorbidities

Patient comorbidities can present challenges in surgical planning; however, with careful management and tailored approaches, high‐quality surgical outcomes are still achievable. From the GEOCC survey, clinicians from all income status countries reported comorbidities to be a common barrier in delivering optimal OC care.^6^ Data from the National Cancer Database (NCDB) showed that 22% of elderly patients over the age of 75 years with advanced OC received only systemic therapy and 23% were left untreated altogether.^40^ Their analysis found that with increasing age and higher comorbidity scores patients were less likely to undergo surgical intervention.^40^ In the survey carried out by Norell et al.,^28^ physicians from Norway found an older population to be less of a barrier for cytoreductive surgery. Interestingly, Norway has one of the highest survival outcomes for older patients with distant metastasis compared to other high‐income countries, likely due to more surgical intervention performed in this patient population.^28^

### 3.3 Socioeconomic factors

Socioeconomic factors play a crucial role in shaping cancer outcomes. The GEOCC thematic analysis identified societal and economic challenges as two major barriers affecting OC treatment.^41^ A systematic review assessing the impact of socioeconomic status (SES) on OC surgery in the UK was conducted by Pickwell‐Smith et al.^42^ The results found that patients from the most deprived areas were significantly less likely to receive surgery compared to those from the least deprived areas (OR range 0.24–0.99), findings that also surely relate to other countries.^42^ Similar findings were also reported in a USA‐based study where epithelial OC cases from the Surveillance, Epidemiology, and End Results database were analyzed.^43^ Jochum et al.^43^ demonstrated that patients living in areas with lower SES had a 25% higher risk of death compared to patients living in areas with the highest SES. Other socioeconomic factors have also been found to influence OC management, including race and insurance status.^40^ Shalowitz et al.^40^ found that black (OR 0.65, 95% CI 0.61–0.70) and Native American (OR 0.65, 95% CI 0.50–0.84) patients had a significantly lower likelihood of receiving surgery compared to white patients. In addition, uninsured patients were the least likely to undergo surgery (OR 0.47, 95% CI 0.43–0.52) compared to insured patients, especially in a healthcare system without strong social support aspects.^40^

### 3.4 Underrepresentation of diversity in clinical trials

Racial disparities in OC care are further exacerbated by the lack of diversity in clinical trials. Clinical trials serve as a crucial pathway for patients to access novel treatments and actively contribute to shaping the future of cancer care. Despite the valuable insights obtained from clinical trials, many are often criticized for their narrow inclusion criteria that fail to reflect the diversity of real‐world populations.^44^ This limitation is particularly important in the context of OC surgery, where outcomes have been shown to vary across different racial groups. Ideally, all clinical trials, including surgical trials, should include participants from a wide range of backgrounds to strengthen external validity and ensure that findings can be generalized to all relevant patient groups. A key concern is the persistent underrepresentation of certain populations and the lack of data stratification by important sociodemographic factors, such as race and ethnicity, in many studies.^44^ This omission hinders understanding of treatment effects in these groups and limits the assessment of clinical outcomes across diverse populations. Consequently, such underrepresentation may contribute to suboptimal treatment strategies among OC patients with different ethnic backgrounds. Greater inclusion of participants from different racial and ethnic backgrounds is critical to identify factors that influence treatment responses and outcomes. To help address this, the Gynecological Cancer Intergroup have released strategies to improve equity, diversity, and inclusion in gynecologic cancer trials that could also be applied to the “real world” treatment landscape.^45^

## 4 PHYSICIAN‐RELATED BARRIERS

### 4.1 Suboptimal surgical training

The multivisceral and multidisciplinary nature of maximal effort cytoreductive procedures presumes and necessitates a highly skilled surgical and perioperative team to ensure the safe delivery of care without increasing complications or negatively affecting a patient's long‐term quality of life.^46^ The GEOCC survey of healthcare professionals found that in LMLI countries extensive resections were less frequently routinely performed during cytoreductive surgery. In addition, a lack of access to radiology, pathology, and genetic services are all reported barriers. In high‐income countries, the main barriers were lack of surgical time, staff, and patient preferences. Here, surgical expertise was a barrier in 9.3% versus 31.9% in high‐income versus LMLI countries, respectively.^6^ The study also highlighted significant variation in the types of resections surgeons were willing to perform for complete cytoreduction.^6^ Rates for large bowel resections were similar between UMI (74.9%) and LMLI (70%) countries but were much higher in high‐income countries (94.9%).^6^ In addition, rates of upper abdominal and diaphragm resections in high‐income countries (76.3% and 82.5%, respectively) were nearly double those in LMLI countries (37.4% and 40%, respectively).^6^ These differences in surgical practices are likely a contributing factor to the survival differences seen in OC patients around the world, shaping the so‐called postcode lottery in oncology care.

In their study on data from the NCDB on 210 667 epithelial OC patients, Shalowitz et al.^40^ reported that 18% of patients had not received any surgery during their OC journey, with 95% of them having high tumor burden. Upon further investigation, 80% of cases had no clear reasoning for why surgery was not performed. A surgical inability to take on and manage those extensive surgeries is presumed, demonstrating how the lack of surgical expertise remains a global problem. In a survey from the Australian and New Zealand gynecology oncology community, 24% of surgeons reported a lack of expertise as a barrier to performing ultraradical surgery.^47^ In addition, 38% of these surgeons who did not perform cytoreductive surgery in the primary setting did so due to a lack of expertise.^24^, ^47^ In South Africa, 55% of surveyed gynecologic oncologists identified a lack of expertise as a key barrier to performing ultraradical surgery.^48^ Confluent peritoneal and diaphragmatic disease were reported to be the most common findings preventing optimal debulking.^48^ This highlights the global challenge of insufficient surgical expertise in managing OC patients with advanced disease.

### 4.2 Ethos, tradition, and surgical philosophy

Apart from technical competency, surgical ethos, tradition, and overall philosophy contribute significantly to the surgical decision‐making process. Despite the well‐defined aims of cytoreductive surgery to achieve total macroscopic tumor clearance, there is a vast variation and discrepancy in clinicians' perceptions, views, and attitudes towards patients' selection as well as limitations of operability and resectability. From the GEOCC survey, it was evident that a large proportion (27.8%) of clinicians from LMLI countries perceived the goal of surgery to be less than 1 cm of residual disease compared to 8.2% in high‐income countries.^6^ This is over a threefold difference.^6^ In addition, 8.5% of clinicians from LMLI countries reported the goal of surgery to be less than 2.5 cm residual disease compared to 2.1% in high‐income countries, a near fourfold difference.^6^ A surgeon's perception of the goals of surgery can directly impact patient care and thus subsequent survival.

A surgeon's understanding and adherence to best practice guidelines can shape their approach to cytoreductive surgery. In the GEOCC survey, nearly 80% of clinicians reported treating over 75% of patients according to international guidelines.^6^ Currently various guidelines exist for the management of OC. To better understand international practices, Norell et al.^28^ focused on comparing guidelines from seven high‐income countries as well as international guidelines, such as those from ESGO. In general, all guidelines were consistent in their recommendations about cytoreductive surgery; however, they differed in the number of revisions made and their last updates.^28^ Adherence to guidelines is essential and should be standard practice for optimal OC care. A study by Bristow et al.^49^ found that hospitals with higher observed adherence to guidelines had greater patient survival outcomes (68.6–79.3 months) compared to those with lower adherence (44.8–50.8 months). Similar findings were demonstrated in the German QS OVAR study, which showed adherence to guidelines to have a prognostic impact on partially platinum sensitive recurrent ovarian cancer.^50^ In a cohort of 233 patients with partially platinum sensitive recurrent ovarian cancer, nearly 50% of patients did not receive standard of care as per national guidelines, resulting in a reduced median overall survival compared to those who received the recommended treatment.^50^

### 4.3 Limited access to training and educational resources

Inadequate access to training resources presents a major obstacle, as it restricts a surgeon's opportunity to improve on cytoreductive techniques and keep up with current best practices. Currently, there is no universally adopted standardized structure for gynecologic oncology training. In fact, in many parts of the world, gynecologic oncology is not recognized as its own subspecialty.^51^ One of the five barriers found in the thematic analysis of the GEOCC survey was the organization of the specialty.^41^ For example, in Brazil, there is no dedicated and approved gynecologic oncology program; instead clinicians must either do general surgery followed by surgical oncology, or general obstetrics and gynecology with only a few months in gynecologic oncology.^24^, ^51^ This issue is not just limited to Latin America. In Europe, clinicians in Bulgaria undergo general gynecology training and then subsequently take up further training in general oncology for gynecologic oncology.^51^ In Italy and Spain, there is also no national accreditation for gynecologic oncology subspecialization. In Africa, training programs have been established for nearly a decade now; however, they face ongoing challenges for further development and expansion due to reliance on external mentorship, lack of resources, and underfunding.^51^ The problem is not limited to only UMI and LMLI countries, but training can also vary in high‐income countries where programs are more well established. Nguyen et al.^52^ performed a clinician survey of 124 graduates from USA‐based gynecologic oncology fellowship programs in the last 6 years to assess the comfort level of in performing certain procedures after fellowship. Interestingly, there was high level of comfort reported for procedures such as open and minimally invasively pelvic lymph node dissection and OC debulking.^52^ However, high rates of discomfort were reported for some ultraradical procedures, such as splenectomy and diaphragmatic stripping.^52^ Variation in training received, skills obtained, and confidence of surgeons can all directly impact a surgeon's ability and willingness to perform surgery for advanced OC, impacting patient care.

### 4.4 Multidisciplinary team patient management

MDT management allows for an expert‐based collaborative approach to patient care, which has been shown to improve outcomes.^53^ Currently, MDT discussion is recommended by various guidelines, including the ESGO quality indicators for advanced OC surgery.^12^ As per ESGO, at a minimum, the MDT for OC should consist of a gynecologic oncology surgeon, a pathologist, a radiologist, and a clinician who can administer chemotherapy.^12^ The GEOCC survey revealed that most clinicians reported having an MDT meeting to discuss OC patients.^6^ However, hospitals in LMLI countries had significantly lower odds of having an MDT at individual hospitals compared to high‐income countries (OR 0.4, 95% CI 0.2–0.8).^6^ In addition, MDT composition varies widely between countries.^6^ Of the clinicians, 25% reported the absence of a radiologist and 20% reported the absence of a pathologist.^6^ Obtaining multiple expert opinions is vital for achieving the best possible treatment, as each expert's insight can influence a patient's care plan.^53^ A deeper analysis on the workings of the MDT was conducted by Khassan et al.^54^ in the UK. Using a validated scoring tool, they assessed MDTs across five cancer alliances and correlated these scores with clinical outcomes.^54^ They observed variations in surgeon participation across MDTs and in one center, linked significantly lower involvement from the surgical team to reduced rate of OC surgery.^54^ An MDT is valuable not only for treatment planning but also during cytoreductive surgery.^55^ Mulligan et al.^55^ reported 5‐year outcomes of OC patients treated with an intraoperative MDT approach and reported increased primary cytoreductive surgery rates, complete tumor clearance, and reduced disease progression. This, once again, highlights the impact collaborative work can have on the delivery of optimal surgical care.

## 5 BRIDGING THE GAP

Recognizing the barriers to optimal surgical care for advanced OC patients is essential for paving the way towards impactful solutions. Large‐scale international initiatives, such as the GEOCC project by the IGCS and EWS by the World Ovarian Cancer Coalition, have significantly advanced our understanding of the challenges that patients and physicians face worldwide in OC care.^6^, ^11^ Overcoming these barriers now requires a coordinated global effort and robust advocacy to ensure equitable access and improve outcomes for patients around the world.

Addressing health system‐related barriers requires collective action from healthcare professionals, government bodies, and patient advocacy groups. For effective centralization of care, centers of excellence must be designated alongside health policy restructuring. International societies, such as ESGO, have established criteria for centers to achieve standardized accreditation or Centre of Excellence status, which can help improve OC care.^12^, ^56^ Healthcare professionals should also aim to implement quality registries and audit practices at their centers to enhance patient care and demonstrate the impact of these improvements on outcomes. Furthermore, patient advocacy groups and healthcare professionals need to continue to raise awareness about OC through education campaigns. This is not only important for patients, but also to help increase funding for resources and clinical research into OC. In countries lacking advocacy groups, societies and healthcare professionals should collaborate with local organizations to establish them.

While accrediting certain centers across each country can help centralize care, it is essential that these centers remain accessible to all patients, not just those who live nearby. Healthcare bodies must establish streamlined referral pathways to ensure specialist access for all patients. Furthermore, telemedicine consultations should be leveraged as a solution for patients with geographic or travel limitations, ensuring access despite these challenges. Telemedicine can also be utilized to conduct regional MDT meetings to discuss patient cases with specialist input. Although patient comorbidities can complicate OC management, prehabilitation and gynecology oncology‐specific Enhanced Recovery After Surgery protocols have improved outcomes, reducing postoperative complications and hospital stays.^57^ Widespread adoption of these pathways should be encouraged to enhance surgical care. Clinicians must be mindful of the impact that SES and race can have on access to care and patient outcomes, ensuring they provide guideline‐adherent care for all patients. Efforts should also be made to increase the diversity of OC patients participating in clinical trials and foster collaborative initiatives with centers in LMLI countries. This would improve patient access to treatments, enhance representation from these regions, and ultimately lead to more generalizable results, bridging the current data gap. In addition, governments must aim to improve funding support for patients who face financial barriers, fostering equitable healthcare access.

Enhancing training and surgical skills for gynecology oncologists is essential to improving OC surgical care. ESGO is working to standardize global training through a defined curriculum, with trainees in accredited programs reporting significantly higher levels of satisfaction, training quality (*P* < 0.0005), and educational environment (*P* = 0.001).^51^ ESGO now also offers a certification exam, which is particularly beneficial in countries where gynecologic oncology is not a recognized subspecialty.^12^, ^51^ To increase opportunities for trainees from UMI or LMLI countries, funded programs for overseas fellowships should be offered through societies like IGCS and ESGO. Subsidized as well as funded opportunities should also be made available for trainees and clinicians to attend international conferences and workshops to further develop skills. Furthermore, physicians from abroad should take the initiative to aid training, mentorship, and the organization of gynecologic oncology programs for LMLI countries.^51^ Experts skilled in ultraradical procedures can play a vital role in addressing the skill gap in these regions. One way to achieve this is by using well‐established gynecologic oncology training programs as models and adapting them to meet the specific needs of LMLI countries. This can further be supported by training, building partnerships, and promoting international collaborations with LMLI physicians. Through shared expertise, physicians in LMLI countries can enhance their surgical skills and improve patient outcomes.

In fulfilling their responsibility, gynecology oncology surgeons must commit to advancing OC care by continuously refining their skills, adhering to best practices, and advocating for equitable, high‐quality treatment options, ensuring all patients receive the best possible outcomes.

## AUTHOR CONTRIBUTIONS

All authors contributed to manuscript design, writing, and review. All authors approved the final version of the manuscript.

## CONFLICT OF INTEREST STATEMENT

S.V. and R.P. have no conflicts of interest. Outside of the submitted work, C.F. reports honoraria received from AstraZeneca, GSK, MSD, Oncoinvent, Ethicon, and Roche. Outside of the submitted work, R.A. reports honoraria received from MSD, EISAI, GSK, and Roche. Outside of the submitted work, M.C.L. reports having a consulting or advisory role for AstraZeneca, Boryung, CKD Pharm, Genexine, Hospicare, GI Innovation, Takeda, and receiving research funding from AbbVie, Amgen, Astellas, AstraZeneca, BeiGene, Cellid, CKD Pharm, Clovis, Eisai, Genexine, GSK, Incyte, Merck, MSD, OncoQuest, Pfizer, and Roche.

## Data Availability

Data sharing is not applicable to this article as no new data were created or analyzed in this study.
